# JIA pathogenesis - genetics

**DOI:** 10.1186/1546-0096-12-S1-I2

**Published:** 2014-09-17

**Authors:** Wendy Thomson

**Affiliations:** 1 Arthritis Research UK Centre for Genetics and Genomics, University of Manchester, Manchester, UK

## 

Juvenile Idiopathic Arthritis is a heterogeneous disease with significant variability in long-term outcome and treatment response; this occurs both between and within JIA subtypes, as defined by the current International League Against Rheumatism (ILAR) classification. There are currently no robust clinical or biological predictors of outcome or treatment response in JIA. Given that JIA is a complex genetic disorder, genetic studies provide an opportunity to address this issue, and whilst previous studies have often been limited by statistical power to identify the likely modest effect sizes, the recent establishment of a number of international consortia for JIA genetics has allowed this issue to be resolved.

This presentation will summarize the current understanding of the genetic basis of JIA susceptibility, prognosis and treatment response and will describe how these genetic associations may further our understanding of the molecular mechanisms and immunological pathways involved in this disease. In addition it will provide insights into how we might utilise this data to progress towards the ultimate goals of predicting long-term disease outcomes at onset, predicting drug response, and move towards more targeted treatment options for children with JIA.

## Disclosure of interest

None declared.

